# Differences in Insula and Pre-/Frontal Responses during Reappraisal of Food in Lean and Obese Humans

**DOI:** 10.3389/fnhum.2016.00233

**Published:** 2016-05-20

**Authors:** Saurabh Kumar, Felicitas Grundeis, Cristin Brand, Han-Jeong Hwang, Jan Mehnert, Burkhard Pleger

**Affiliations:** ^1^Max Planck Institute for Human Cognitive and Brain SciencesLeipzig, Germany; ^2^Day Clinic for Cognitive Neurology, University Hospital LeipzigLeipzig, Germany; ^3^Kumoh National Institute of Technology, Department of Medical IT Convergence EngineeringGumi, South Korea; ^4^Department of System Neuroscience, Universal Medical Center Hamburg-EppendorfHamburg, Germany; ^5^Department of Neurology, BG University Clinic Bergmannsheil, Ruhr-University BochumBochum, Germany

**Keywords:** obesity, EEG, dorsolateral prefrontal cortex, frontal operculum, insular cortex, reappraisal of food

## Abstract

Brain regions involved in the reappraisal of tasty but unhealthy foods are of special interest for the development of new therapeutic interventions for obesity, such as non-invasive brain stimulation or neurofeedback. Here, we visually presented food items (i.e., high/low caloric) to obese and lean individuals during electroencephalogram (EEG) recordings, while they either admitted or regulated their food desire. During admitting the desire for low and high calorie foods, obese as well as lean individuals showed higher activity in the left dorsolateral prefrontal cortex (DLPFC), whereas the right frontal operculum was involved in the reappraisal of the same foods, suggesting interplay between executive control and gustatory regions. Only in lean participants, we found an interaction between calorie content and the regulate/admit conditions in bilateral anterior insular cortices, suggesting that the anterior insula, assumed to primarily host gustatory processes, also underpins higher cognitive processes involved in food choices, such as evaluating the foods’ calorie content for its reappraisal.

## Introduction

Obesity is a major health burden and dramatically climbing incidence rates, especially in rapidly developing countries like China or India, lead to a demand on developing new therapeutic strategies (Roman et al., 2014). Currently available weight-loss programs consist either of dieting (Soeliman and Azadbakht, 2014), physical activity (Jakicic and Davis, 2011), or the combination of both (Amorim Adegboye and Linne,2013), with mostly modest and also timely restricted effects on participants’ body weight. The majority of participants start regaining weight directly after the program has ended. Based on these experiences, new therapeutic strategies started combining dieting and physical activity with regular psychological interventions to strengthen motivation and volition (Looney and Raynor, 2013; Ausburn et al., 2014). This combination seems specifically effective for stabilizing the program-associated weight-loss beyond the program’s duration, but the influences on body weight and metabolism are *per se* small. Establishing new programs that, at the same time, produce profound weight loss and long-term body-weight stability seem generally difficult because the neuronal mechanisms driving and sustaining overeating are still not well understood.

Regular consumption of high-calorie foods affects the brain’s reward system in comparable ways as addictive drugs (Volkow et al., 2013). If rats consume such foods over several weeks, they react with compulsive eating habits that resemble drug craving behavior (Johnson and Kenny, 2010). One mechanism driving this addiction-like behavior is an altered dopaminergically mediated reward response to foods (Wang et al., 2001; Stice et al., 2008). Regular consumption of high-calorie foods regularly amplifies the dopaminergic response in regions underpinning habitual eating behavior such as the dorsal striatum, which over time is compensated by a reduction of the striatal dopamine receptor availability (Stice et al., 2008; Johnson and Kenny, 2010; Volkow et al., 2013). In rats, this reduced receptor availability leads to weakened dopaminergic responses to the same foods as before high-calorie diet, which, in turn, supports further overeating (Johnson and Kenny, 2010).

Wanting food is different from liking food, but both together are necessary for food-related reward responses (for a review see Berridge, 2009). Wanting food without liking it is merely a sham or partial reward, without the gustatory and olfactory pleasure. “Wanting” is still an important component of food reward, especially when combined with “liking”. Food reward cannot happen without incentive salience, even if hedonic “liking” is present. Hedonic “liking” by itself is simply a triggered affective state. It is the process of incentive salience attribution that makes a specific associated food the object of desire. “Liking” and “wanting” are needed together for full food reward. Fortunately, both usually happen together in human life (Berridge, 2009).

Brain regions involved in the reappraisal of wanting and liking food are of special interest since the modulation of their functional implementation within brain circuitries commonly orchestrating eating behavior may represent a future target for brain-stimulation or neurofeedback training. Whether such interventions underpin, accelerate, or even initiate changes in body weight remains another area for future research.

On the search for neurofeedback targets, we recently used functional magnetic resonance imaging (fMRI) in a group of lean to overweight participants to identify brain regions involved in the reappraisal of food (Hollmann et al., 2012). As in the present study, participants were visually presented food items under two different conditions: Either they admitted the desire for the presented food by thinking, e.g., of its taste and flavor (i.e., admit condition), or they regulated their desire by thinking, e.g., that the food is unhealthy or its consumption is followed by weight gain (i.e., regulate condition). Comparing the regulate to the admit condition, we identified the dorsolateral prefrontal cortex (DLPFC), pre-supplementary motor area (pre-SMA) and inferior frontal gyrus (IFG); regions that are well known to underpin top-down control of craving, inhibition of learned associations and prepotent responses. Furthermore, we observed increased activation in bilateral OFC, a key region of the brain’s reward valuation system, as well as the anterior insula together with the frontal operculum and temporoparietal junction (TPJ) suggesting interoceptive awareness and self-reflection. These results suggest that reappraisal of food recruits the brain’s valuation system in combination with prefrontal cognitive control areas associated with response inhibition (Hollmann et al., 2012).

FMRI is one method to assess neural underpinnings in the cortex. These neural responses can also be acquired in real-time for neurofeedback training. Real-time fMRI for neurofeedback training, however, bears several limitations, such as the spatial (i.e., magnetic resonance imaging (MRI) environment), application-based (i.e., no self-application) and temporal (i.e., limited MRI scanning time, latency of the hemodynamic response) restrictions. Many fMRI-based neurofeedback attempts therefore failed in translating the training effect into every-day behavior. Electroencephalogram (EEG) instead offers real-time feedback capability, longer training and self-application, despite lower spatial brain resolution. In the present study, we therefore used EEG in combination with a study design adapted from our recent fMRI study (Hollmann et al., 2012), to identify neuronal responses involved in regulating the desire for food in obese as well as lean individuals. We hypothesized, that comparing EEG responses of the regulate and admit condition reveals neuronal activation in brain areas involved in executive control and active reappraisal, such as the DLPFC in the prefrontal cortex. Furthermore, we expected differences in the DLPFC’s activity for lean as compared to obese participants, as well as for visually presented high as compared to low calorie foods.

## Materials and Methods

### Participants

This study was approved by the local Ethics Committee of the Medical Faculty Leipzig and carried out according to the Declaration of Helsinki. All participants gave written informed consent prior to their participation. Forty-six right-handed participants took part in this study. Half of them were lean (Body mass index (BMI) >20 and <25 kg/m^2^, mean = 23, *SD* = 1.4) and the other half obese (BMI > 30 kg/m^2^, mean = 36.81, *SD* = 6.21). Participants were financially reimbursed for their participation. All participants were fasting for 5 h before the experiment and were tested around noon (12 am to 2 pm). As compared to shorter fasting periods, 5 h enhanced participants’ attention for the visually presented food items as well as their effort in regulating their food desire (pilot data, not shown). Exclusion criteria were any present or past neurological or psychiatric diseases, as well as prescribed central acting drugs. Depression was assessed using the BDI-II questionnaire. A score of 29 or higher, indicating severe depression, was considered as an exclusion criterion. Five participants had to be excluded due to high BDI-II scores. One other subject had to be excluded due to technical problems in data recording. The remaining 40 subjects consisted of 20 males and 20 females. Each gender group consisted of 10 lean and 10 obese participants (see Table 1). Groups were matched for age (unpaired *t*-test *p* > 0.5). In addition, the BMI did not differ between males and females (*p* > 0.1), neither for the group of lean (*p* > 0.3), nor for obese participants (*p* > 0.2).

**Table 1:** **Mean and standard deviation (SD) for age and body mass index (BMI) for the four different study groups**.

N/subgroup	Age range (mean) [years] ± (SD)	BMI range (mean) [kg/m^2^] ± (SD)
10 lean males	24–33 (29.2) ± (3.3)	20.6–24.8 (23.1) ± (1.3)
10 lean females	25–34 (28.6) ± (3.3)	20–24.9 (22.5) ± (1.5)
10 obese males	23–37 (28.6) ± (4.2)	30.9–55 (38.3) ± (7.3)
10 obese females	23–33 (27.9) ± (2.9)	31.4–42.9 (35.3) ± (4.0)

**Figure 1:**
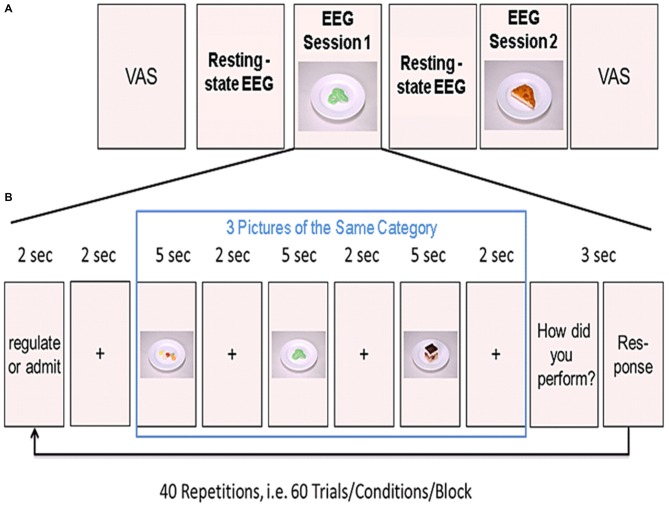
**Experimental design. (A)** Shown is the order of the two resting-state and the two experimental sessions. Before the first resting-state electroencephalogram (EEG) measurements, participants rated their tiredness, hunger, satiety, thirstiness, and stress levels on a visual analog scale (VAS). **(B)** Example of one block from the task-based EEG session. Three food pictures from one category (high or low calorie) were presented in a row. Prior to the presentation of the pictures, participants were instructed to either regulate or admit their desire for the upcoming food pictures. After presentation of the three pictures, participants rated their ability to either regulate or admit the desire for the three presented foods. This was repeated 20 times for each condition (regulate/admit), resulting in a total of 40 blocks.

### Visual Analog Scales (VAS)

By means of a quasi-continuous, digital VAS, we assessed six psychological states before and after the experiment. The processed levels for each VAS ranged from 0 on the left hand side (i.e., not at all) to 100 on the right hand side (i.e., fully true) and the following questions were raised: “How tired are you?”, “How hungry are you?”, “How dry is your mouth?”, “How stressed do you feel?”, “How thirsty are you?”, “How sated are you?” For pre-post comparisons, we used the paired *t*-test.

### EEG Recording

EEG data was recorded with a 64-channel Brain Amp recorder (Brain Products, Gilching, Germany) with 1000 Hz temporal resolution. We applied 63 electrodes apart from the reference and the ground electrodes to participant’s scalp arranged according to the international 10–10 system. One additional electrode was attached below the left eye to measure vertical eye movements (i.e., electro-occulogram or EOG).

### Experimental Schedule

Participants were comfortably seated in front of a computer screen in a shielded EEG cabinet. First, we acquired 5 min of task-free resting-state EEG data to familiarize participants with the environment. These resting-state measurements were followed by the first session of task-based EEG recordings (20 min). Afterwards, we acquired another 5 min resting-state data. Participants were asked to relax during these 5 min. Finally, we recorded a second session of task-based EEG (see Figure 1A).

### Task-Based EEG Recordings

During EEG recordings, we presented food pictures on the computer screen in front of the participant. Before the experiment, participants were instructed to either admit their desire for the presented food (ADMIT condition) or to regulate their desire (REGULATE condition) according to the instructions presented on the computer screen. The trials were grouped into blocks of three trials. At the beginning of each block, an instruction was shown. The instruction on the screen was either “ADMIT” or “REGULATE”. The instruction was presented for 2 s followed by a crosshair for another 2 s. Afterwards, we presented the three food pictures. Each picture was presented for 5 s followed by a crosshair for 2 s (see Figure 1B). The order of blocks was pseudo-randomized across each session.

We used a 2-by-2 factorial design with the factors REGULATE/ADMIT-by-high/low calorie foods. Each calorie group consisted of an equal amount of sweet and savory foods. The comparison of sweet to savory (and *vice versa*) was of no interest and only implemented to better meet participants’ food preferences and to keep the task interesting. Sixty food pictures were chosen from a pre-rated in-house repertoire of standardized food pictures with 60 pictures for each condition (Hollmann et al., 2012). In each of the 2 experimental sessions, we acquired 20 blocks (three food pictures per block) for both, the ADMIT and the REGULATE condition. To cancel out the influence of the presented foods, each food picture was presented twice, one time in the REGULATE, and the other time in the ADMIT condition in each session. After presenting the three food pictures, another screen with a 4-point Likert scale showed up for 3 s and participants rated their subjective impression on how well they regulated or admitted their desire for the three food items. The scale of these self-ratings were ranged from 1 (very bad) to 4 (very good).

After the experiment, we asked participants about the specific mental strategies they used to either regulate or admit their desire for the presented food items. The different strategies are summarized in Table 2.

### Preprocessing of the EEG Data

Using the FieldTrip Software package (Donders Centre for Cognitive Neuroimaging, University Nijmegen, Netherlands) and the Berlin Brain Computer Interface (BBCI) toolbox (Berlin Institute of Technology, Germany), EEG data was first down-sampled to 250 Hz and band-pass filtered (3rd order Butterworth filter) between 0.05 and 45 Hz (BBCI toolbox). Then the data was re-referenced from the original reference of FCz to the common average reference (CAR; Bertrand et al., 1985; Pascual-Marqui and Lehamann, 1993). To correct for eye movement and facial muscles contractions we regressed out the sum of recordings from channels Fp1 and Fp2, indicating horizontal EOG, and the subtraction of channels Fp1 and EOG indicating vertical EOG, respectively, with a least mean-square fitting procedure (Parra et al., 2005). Since these channels acted as EOG channels, they were rejected from further analysis.

Thereafter, the EEG data was epoched into trials of 5 s length (i.e., presentation time for one food item) and baseline corrected using the mean value of the time course for the particular trial. The self-ratings across all epochs (i.e., same value for each picture within one block) were added as an interacting covariate. The temporal window of interest was identified by a heuristic search, based on a signed point-biserial correlations that has been widely used in event-related potential (ERP) based brain-computer interfaces (BCIs) to select the most discriminative temporal windows between different experimental conditions (Blankertz et al., 2011). In particular, the sums of the absolute correlation coefficient values at the given time window were calculated and then the temporal window corresponding to the highest sum value was selected for the analyses. The time period between 1675 and 2055 ms after stimulus onset was selected for source localization.

**Table 2:** **Mental strategies the participants used in order to admit or regulate their desire of the foods**.

ADMIT	Obese (*N* = 20, 28 indications)	Lean (*N* = 20, 27 indications)
Imagination of consuming	14	7
Combination with other food	6	9
Positive environment/atmosphere	3	1
Positive properties of the food	2	5
Appetite	0	3
Other/none specific strategy	3	2
**REGULATE**	Obese (*N* = 20, 27 indications)	Lean (*N* = 20, 25 indications)
Negative properties (rotten, etc.,)	12	11
Suppression of thinking about	8	2
Persuade oneself of being sated	1	8
Consequences for ones body	2	1
Other	4	3

*Note that some participants used more than one strategy during the course of the experiment. That is why the N is higher than the actual number of participants*.

### Source Localization of the EEG Data

Source localization was done with the Statistical Parametric Mapping (SPM) Software package 12 (Wellcome Trust Centre for Neuroimaging at University College London, UK,^1^), running under MATLAB version 8.2 (The MathWorks, Ismaning, Germany). The forward model consists of the model of the brain itself, which was formed by the boundary element method (BEM) with the different layers of the brain tissue, the skull and the scalp. The co-registration was done by matching the electrode sensor locations on participant’s scalp and the coordinate mapping from the scalp to the cortex. The standardized MRI was used with this cortical mesh model as implemented in SPM12. The inverse problem was solved by the multivariate source pre-localization (MSP) algorithm (Mattout et al., 2005).

**Figure 2:**
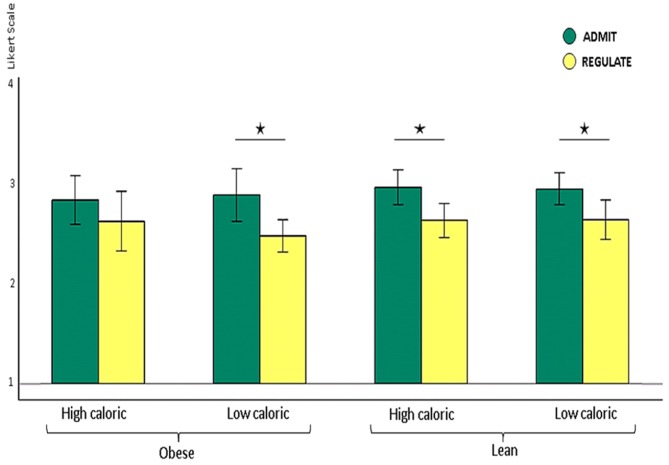
**Shown are lean and obese participants’ self-ratings on how well they either admitted or regulated the desire for high and low calorie foods on the Likert scale from 1 (very bad) to 4 (very good) shown as *y*-axis**. Lean participants rated their ability to admit the desire for foods as better than their ability to reappraise the foods, irrespectively of whether this was high (*p* < 0.009) or low caloric (*p* < 0.032). Obese participants also rated their performance better for admitting the desire for low calorie foods (*p* < 0.007). For high calorie foods, however, they unexpectedly rated their performance equally well (*p* > 0.2). The whiskers index the standard errors and the significance is marked with asterisks.

On the group level we used the full factorial design as implemented in SPM 12 with the independent factor “obese/lean participants”, and the dependent factors “high/low calorie foods” as well as “REGULATE/ADMIT”. A family-wise error (FWE) corrected *p*-value of < 0.05 together with a minimum cluster size to 20 voxel indicated significance. We used *post hoc* paired (within-subject) and unpaired (between-subject) *t*-tests to decipher the structure of significance.

**Figure 3:**
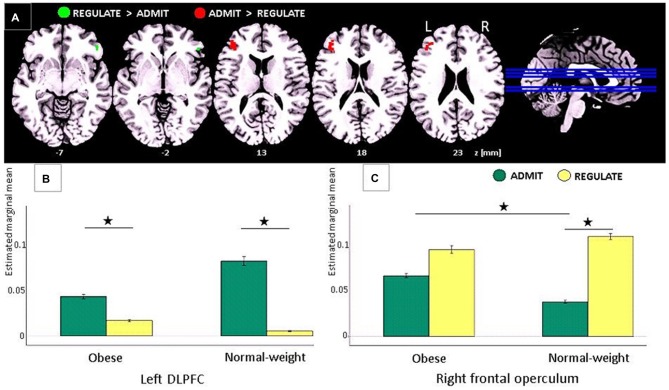
**Task-based EEG results. (A)** ADMIT > REGULATE (i.e., interaction between self-ratings and EEG activity) across lean and obese participants resulted in activation of the left dorsolateral prefrontal cortex (DLPFC; red cluster, family-wise error (FWE)-corrected, *p* < 0.05). REGULATE > ADMIT activated the right frontal operculum (green cluster, FWE-corrected, *p* < 0.05). “*z*” Indicates the MNI coordinates of the axial brain slices. “R” indicates the right and “L” the left brain hemisphere. **(B)** Bar plots show the estimated marginal means for REGULATE and ADMIT in the left DLPFC separately for lean and obese participants. **(C)** Bar plots show the estimated marginal means for REGULATE and ADMIT in the right frontal operculum separately for lean and obese participants. For **(B,C)** the whiskers index the standard errors and significance is marked with asterisks.

### Analyses of the Self-Ratings Assessed During the EEG Experiment

Besides the implementation of the trial-by-trial self-ratings as a covariate for the single participant EEG analyses, we also applied them to an ANOVA with the dependent factors “REGULATE/ADMIT”, and “high/low calorie foods”, as well as the independent factor “obese/lean participants” (same model as for the analyses of the EEG data). In case of significance, we applied *post hoc* paired (within-subject) and unpaired (between-subject) *t*-tests to investigate the structure of significance.

## Results

### Visual Analog Scales

Comparing the post VAS to the pre VAS, we found significantly increased rating for tiredness (paired *t*-test *p* < 0.001), and hunger (*p* < 0.001), whereas the satiety ratings significantly decreased (*p* < 0.035).

### Self-Ratings in the REGULATE/ADMIT Conditions

We found higher self-rating scores for the ADMIT as compared to the REGULATE condition for lean and obese participants together, across high and low calorie foods (ANOVA, *p* < 0.0001). *Post hoc* paired *t*-tests revealed that lean participants rated their performance better for admitting, relative to regulating their desire for food, irrespectively of whether this was high (*p* < 0.001) or low caloric (*p* < 0.009). Obese participants also rated their performance better for admitting, relative to regulating their desire for low calorie foods (*p* < 0.007). For high calorie foods, however, they unexpectedly rated their performance equally well (*p* > 0.2; Figure 2).

### Task-Based EEG Findings

Comparing the ADMIT to the REGULATE condition (i.e., interaction between self-ratings and EEG activity) for both, lean and obese participants, we found a FWE-corrected activation (*p* < 0.05) in the left DLPFC (peak voxel: MNI coordinates (*x, y, z*): −42, 38, 20 mm, *T* = 5.55, *p* < 0.0005; *post hoc* paired *t*-tests for lean *p* < 0.005 and for obese *p* < 0.012), whereas the inverse contrast (REGULATE > ADMIT), revealed an FWE-corrected activation in the right frontal operculum (peak voxel: MNI coordinates (*x, y, z*) 50, 34, −6 mm, *T* = 5.28, *p* = 0.0123; see Figure 3A). According to *post hoc* paired *t*-tests, the latter effect was driven solely by lean participants (*p* < 0.001; obese group *p* > 0.137; see Figure 3C). Comparing both study groups, we furthermore found higher activations in the ADMIT condition in obese as compared to lean participants (*p* < 0.04; Figure 3B).

**Figure 4:**
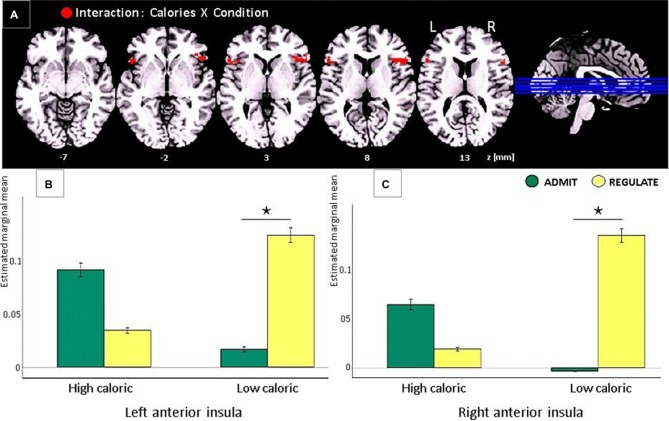
**Task-based EEG results of the interaction between “calorie content” (high-caloric/low-caloric) and “condition” (admit/regulate, including the self-ratings as covariates) in lean participants only**. Note that in obese participants the same comparison revealed no significant effects. **(A)** In lean participants we found activations in the anterior insular cortex in both hemispheres (red clusters, FWE corrected, *p* < 0.05). **(B)** Bar plots show the estimated marginal means for high and low calorie foods for either the REGULATE or the ADMIT condition in the left anterior insula. **(C)** Bar plots show the estimated marginal means in the right anterior insula, respectively. The whiskers indicate the standard errors and significance is marked with asterisks.

Only lean participants showed a significant interaction between “calorie content” (high-caloric/low-caloric) and “condition” (ADMIT/REGULATE), not in the DLPFC or the frontal operculum, but in the anterior insular cortex of both hemispheres (left: −50, 20, 8, *T* = 6.45, *p* < 0.001; right: 50, 22, 8, *T* = 6.32, *p* < 0.0003; Figure 4A). *Post hoc* *t*-tests revealed a significantly higher activity of the insula during the REGULATE as compared to the ADMIT condition in the left (*p* < 0.01) and right insular cortex (*p* < 0.001), but only for low calorie foods. For high calorie foods, we found the opposite pattern: higher activations during the ADMIT as compared to the REGULATE condition, although the difference between ADMIT and REGULATE did not reach significance (left insula *p* < 0.09, right insula *p* < 0.14; Figures 4B,C).

## Discussion

In agreement with our a-priori hypotheses, we show that in obese as well as in lean individuals, the left DLPFC underpins the desire for foods, however, irrespectively of whether these were high or low caloric. We further hypothesized an interaction between participants’ self-rated ability to reappraise foods and body weight in the DLPFC, which we could not confirm. Not the DLPFC, but the frontal operculum on the contralateral right hemisphere was involved in the self-rated ability to reappraise foods, again irrespectively of calorie content. During admitting the desire for low calorie foods (i.e., interaction between self-ratings and EEG activity), not the frontal or prefrontal cortex, but both hemispheres’ anterior insular cortices responded with high activity for high calorie foods and low activity for low calorie foods. During the reappraisal of the same foods, the same areas showed the inverse pattern: low activity for high calorie foods and high activity for low calorie foods. Nonetheless, the difference between the admit and regulate condition reached significance only for low, and not for high calorie foods.

During EEG recordings, we visually presented food items out of two different categories (i.e., high/low caloric). Each picture was presented twice—one time, participants were instructed to regulate the desire for the presented foods, the other time, they had to allow the desire for the same foods. After picture presentation, participants rated their ability to either regulate or admit the desire for the presented foods on a 4-point Linkert-scale ranging from 1 (very bad) to 4 (very good). These self-ratings were implemented into the EEG data analyses as covariates to assess the related brain activity for either the admit or regulate condition. Participants were free in choosing the best strategy in order to either admit or regulate their food desire. After the experiment, we asked them which strategy they used. For regulating their desire, most participants reported of thinking that the presented foods were rotten. To allow the desire for the same foods they simply thought of its delicious taste during consumption. Comparing these two conditions, irrespectively of whether participants were obese or lean or whether the food was high or low caloric, we found a sub-region within the right frontal operculum involved in the regulation of the food desire. Its activity during admitting food desire was significantly higher in obese as compared to lean individuals possible indicating a stronger gustatory response to the visually presented foods. In the DLPFC on the opposite hemisphere, we found a region involved in the desire for the same foods. During admitting food desire, its activity was enhanced by trend in lean as compared to obese participants probably suggesting a stronger executive control. Together, these findings address opposing effects in food choices to sub-regions within left prefrontal and right frontal cortex in both, lean and obese individuals.

Previous fMRI studies on central nervous taste processing showed, that taste stimuli applied either to the right or left side of the tongue predominantly activate ipsilateral brain regions as well as their connections, not only at the thalamus level, but also in higher-level gustatory cortices (Iannilli et al., 2012). We in the present study, however, found clearly lateralized effects for admitting or regulating food desire in the left DLPFC and right frontal operculum, respectively. In previous fMRI studies using almost the same experimental design as in the present study, we also found that regulating the desire for food activated the frontal operculum, however, not only in one but both brain hemispheres (Hollmann et al., 2012). This suggests that different study groups with different BMI ranges (lean to overweight in Hollmann et al., 2012 vs. lean and obese in the present study) induce differentially lateralized brain effects related to the reappraisal of foods.

Only in lean individuals, we found an interaction between high and low calorie foods, in a region neighboring the frontal operculum, namely the anterior insular cortex. Regarding the insula’s cognitive implementation, recent studies suggest that its activation relates to the sense of ownership and agency (Farrer et al., 2003), or the subjective awareness and affective processing of bodily signals (Craig, 2002, 2004). Especially the anterior insula is assumed to play a major role in viscerosensory (Oppenheimer et al., 1992) and interoceptive cognition (for a review see Craig, 2009), suggesting its involvement in higher-order perceptual processing of the body that is either related to a sense of ownership or to emotional experience. In the context of eating, the anterior insula, together with the neighboring frontal operculum, are described to host the primary gustatory cortex, which is assumed to primarily code taste (Rolls et al., 1988; Zatorre et al., 1992; Small et al., 1999). During eating, exteroceptive food-related sensory signals from taste and olfactory receptor cells activate the anterior insula together with the frontal operculum, where stimulus identity and intensity are merged into a stable representation, independent of the homeostatic or motivational state (Rolls et al., 1988; Zatorre et al., 1992; Small et al., 1999).

Our findings extend these functions, since in lean participants visually presented foods activated the anterior insular cortex, independently of signals from peripheral taste or olfactory receptor cells. This finding is well in line with previous studies in mice, showing that the insular cortex regulates food choices even in the absence of peripheral taste inputs (Oliveira-Maia et al., 2012). One possible interpretation of this finding is that the anterior insula in humans also contributes to the ability to imagery food and taste (as indexed by the self-ratings), with, however, different response profiles for high as compared to low calorie foods. Whether these calorie-related differences in neural responses primarily originate from the insular cortex or mirror top-down influences from other brain sources not captured by EEG remains an open question for future research. Food and taste evaluation and imagery, nonetheless, is an essential function for survival. Its implementation in the primary gustatory cortex may therefore represent an evolutionary well-preserved effect.

Lean and obese individuals together rated their ability to admit the desire for low-calorie foods as better than the ability to reappraise the desire for the same foods. These findings suggest that following the hedonic feelings of wanting and liking foods is easier than their reappraisal (Berridge, 2009). However, only in lean participants, self-ratings were well reflected by the activity obtained from the anterior insular cortices. For high-calorie foods, they showed an inverse insula response profile as for low calorie foods, which, however, did not reach significance. Obese participants unexpectedly rated their ability to reappraise high calorie foods as equally well as the ability to admit the desire for the same foods. However, contrarily to lean participants, self-ratings in obese individuals were not reflected by neuronal responses neither by the insula’s activity levels, nor by any other EEG sources throughout the brain. Although it is problematic to interpret such non-significant effects, since they still may become significant with increasing the sample size, they probably point to an association between obesity and an impaired self-reflection of the ability to reappraise foods in the insular cortex. However, in disagreement with our a-priori hypotheses, we found no differences between groups of obese and lean individuals: a lack of effect, which is possibly driven by the food pictures that we applied in the present study.

These pictures were chosen from a larger assembly that was validated in pilot experiments in only lean individuals (others than those who participated in the present study; data not published). The pictures chosen for the present study were those with the highest ratings in terms of esthetic and tastiness in the photographic presentation. Due to the validation in only lean individuals, the present set of food pictures may have been more sensitive to changes in lean ones, possibly explaining the lack of interaction effects in obese participants (admit/regulate-by-low/high calorie food) as well as the lack of differences between lean and obese participants.

In summary, we show distinct brain regions in obese and lean individuals involved in the evaluation of the food’s calorie content and its reappraisal. The interplay between the left DLPFC and the right frontal operculum may in future serve as a target for non-invasive brain stimulation or neurofeedback studies that aim at modulating eating behavior towards better reappraisal capacities for foods. The involvement of the anterior insular in lean subjects suggests that the insula, so far assumed to host primary gustatory processes, also plays a role in processes underpinning higher cognitive functions involved in food choices.

## Author Contributions

Conception and design of study: SK, JM, BP. Acquisition of data: FG, CB. Analysis and/or interpretation of data: SK, H-JH, FG, CB, JM, BP. Drafting the manuscript: SK, H-JH, JM, BP. Revising the manuscript critically for important intellectual content: SK, H-JH, JM, BP. Approval of the version of the manuscript to be published : SK, FB, CB, H-JH, JM, BP.

## Conflict of Interest Statement

The authors declare that the research was conducted in the absence of any commercial or financial relationships that could be construed as a potential conflict of interest. The reviewer SAS and handling Editor declared their shared affiliation, and the handling Editor states that the process nevertheless met the standards of a fair and objective review.

## Footnotes

^1^http://www.fil.ion.ucl.ac.uk/spm

## References

[B27] Amorim Adegboye A. R. Linne Y. M. (2013). Diet or exercise, or both, for weight reduction in women after childbirth. Cochrane Database Syst. Rev. 7:CD005627. 10.1002/14651858.CD005627.pub3 23881656 PMC9392837

[B1] Ausburn T. F. LaCoursiere S. Crouter S. E. McKay T. (2014). Review of worksite weight management programs. Workplace Health Saf. 62, 122–126. 10.3928/21650799-20140219-06 24811698

[B2] Berridge K. C. (2009). ‘Liking’ and ‘wanting’ food rewards: brain substrates and roles in eating disorders. Physiol. Behav. 97, 537–550. 10.1016/j.physbeh.2009.02.044 19336238 PMC2717031

[B3] Bertrand O. Perrin F. Pernier J. (1985). A theoretical justification of the average reference in topographic evoked potential studies. Electroencephalogr. Clin. Neurophysiol. 62, 462–464. 10.1016/0168-5597(85)90058-9 2415344

[B4] Blankertz B. Lemm S. Treder M. Haufe S. Müller K. R. (2011). Single-trial analysis and classification of ERP components–a tutorial. Neuroimage 56, 814–825. 10.1016/j.neuroimage.2010.06.048 20600976

[B7] Craig A. D. (2002). How do you feel? Interoception: the sense of the physiological condition of the body. Nat. Rev. Neurosci. 3, 655–666. 10.1038/nrn894 12154366

[B6] Craig A. D. (2004). Human feelings: why are some more aware than others? Trends Cogn. Sci. 8, 239–241. 10.1016/j.tics.2004.04.004 15165543

[B5] Craig A. D. (2009). How do you feel–now? The anterior insula and human awareness. Nat. Rev. Neurosci. 10, 59–70. 10.1038/nrn2555 19096369

[B8] Farrer C. Franck N. Georgieff N. Frith C. D. Decety J. Jeannerod M. (2003). Modulating the experience of agency: a positron emission tomography study. Neuroimage 18, 324–333. 10.1016/s1053-8119(02)00041-1 12595186

[B9] Hollmann M. Hellrung L. Pleger B. Schlögl H. Kabisch S. Stumvoll M.. (2012). Neural correlates of the volitional regulation of the desire for food. Int. J. Obes. (Lond) 36125, 648–655. 10.1038/ijo.2011.125 21712804

[B10] Iannilli E. Singh P. B. Schuster B. Gerber J. Hummel T. (2012). Taste laterality studied by means of umami and salt stimuli: an fMRI study. Neuroimage 60, 426–435. 10.1016/j.neuroimage.2011.12.088 22245354

[B11] Jakicic J. M. Davis K. K. (2011). Obesity and physical activity. Psychiatr. Clin. North Am. 34, 829–840. 10.1016/j.psc.2011.08.009 22098807

[B12] Johnson P. M. Kenny P. J. (2010). Dopamine D2 receptors in addiction-like reward dysfunction and compulsive eating in obese rats. Nat. Neurosci. 13, 635–641. 10.1038/nn.2519 20348917 PMC2947358

[B13] Looney S. M. Raynor H. A. (2013). Behavioral lifestyle intervention in the treatment of obesity. Health Serv. Insights 6, 15–31. 10.4137/hsi.s10474 25114557 PMC4089816

[B14] Mattout J. Pélégrini-Issac M. Garnero L. Benali H. (2005). Multivariate Source Prelocalization (MSP): use of functionally informed basis functions for better conditioning the meg inverse problem. Neuroimage 26, 356–373. 10.1016/j.neuroimage.2005.01.026 15907296

[B15] Oliveira-Maia A. J. de Araujo I. E. Monteiro C. Workman V. Galhardo V. Nicolelis M. A. (2012). The insular cortex controls food preferences independently of taste receptor signaling. Front. Syst. Neurosci. 6:5. 10.3389/fnsys.2012.00005 22403530 PMC3290770

[B16] Oppenheimer S. M. Gelb A. Girvin J. P. Hachinski V. C. (1992). Cardiovascular effects of human insular cortex stimulation. Neurology 42, 1727–1732. 10.1212/wnl.42.9.1727 1513461

[B17] Parra L. C. Spence C. D. Gerson A. D. Sajda P. (2005). Recipes for the linear analysis of EEG. Neuroimage 28, 326–341. 10.1016/j.neuroimage.2005.05.032 16084117

[B18] Pascual-Marqui R. D. Lehamann D. (1993). Topographic maps, source localization inference and the reference electrode: comments on a paper by Desmedt et al. Electroencephalogr. Clin. Neurophysiol. 88, 532–533. 10.1016/0168-5597(93)90043-o 7694840

[B19] Rolls E. T. Scott T. R. Sienkiewicz Z. J. Yaxley S. (1988). The responsiveness of neurones in the frontal opercular gustatory cortex of the macaque monkey is independent of hunger. J. Physiol. 397, 1–12. 10.1113/jphysiol.1988.sp016984 3411507 PMC1192108

[B20] Roman S. Agil A. Peran M. Alvaro-Galue E. Ruiz-Ojeda F. J. Marchal J. A.. (2014). Brown adipose tissue and novel therapeutic approaches to treat metabolic disorders. Transl. Res. 165, 464–479. 10.1016/j.trsl.2014.11.002 25433289

[B21] Small D. M. Zald D. H. Jones-Gotman M. Zatorre R. J. Pardo J. V. Frey S.. (1999). Human cortical gustatory areas: a review of functional neuroimaging data. Neuroreport 10, 7–14. 10.1097/00001756-199901180-00002 10094124

[B22] Soeliman F. A. Azadbakht L. (2014). Weight loss maintenance: a review on dietary related strategies. J. Res. Med. Sci. 19, 268–275 24949037 PMC4061651

[B28] Stice E. Spoor S. Bohon C. Small D. M. (2008). Relation between obesity and blunted striatal response to food is moderated by TaqIA A1 allele. Science 322, 499–452. 10.1126/science.1161550 PMC2681095 18927395

[B24] Volkow N. D. Wang G. J. Tomasi D. Baler R. D. (2013). The addictive dimensionality of obesity. Biol. Psychiatry 73, 811–818. 10.1016/j.biopsych.2012.12.020 23374642 PMC4827347

[B25] Wang G. J. Volkow N. D. Logan J. Pappas N. R. Wong C. T. Zhu W.. (2001). Brain dopamine and obesity. Lancet 357, 354–357. 10.1016/s0140-6736(00)03643-6 11210998

[B26] Zatorre R. J. Jones-Gotman M. Evans A. C. Meyer E. (1992). Functional localization and lateralization of human olfactory cortex. Nature 360, 339–340. 10.1038/360339a0 1448149

